# Understanding the small print: a 5-year review of readability in phase I trial information leaflets

**DOI:** 10.1186/s13063-026-09889-9

**Published:** 2026-07-09

**Authors:** Kate Dodd, Tim Rowland, Hassan Burhan, Amin Sammour, Richard J. FitzGerald, Lauren E. Walker

**Affiliations:** 1 NIHR Liverpool CRF, Liverpool University Hospitals NHS Foundation Trust, Liverpool, UK; 2 Centre for Experimental Therapeutics (TherEx), University of Liverpool, Liverpool, UK; 3 University of Liverpool, Liverpool, UK

**Keywords:** Literacy, Participant information leaflets, Phase I, Readability, Reading age

## Abstract

**Background:**

Understanding trial information is essential for participants to give truly informed consent. Current guidance suggests that health information should be accessible to readers with a reading age of 11 years. Readability describes how easily text can be understood, while reading age represents the minimum age at which a typical reader is likely to be able to understand the material. This study examines the readability and reading age of participant information leaflets (PILs) used in recent phase I clinical trials to assess how well they align with recommended standards.

**Methods:**

This single-centre study analysed phase I participant information leaflets (PILs) produced between 2017 and 2022. Five established readability tools were used to assess readability and reading age: Flesch Reading Ease, Flesch-Kincaid Grade Level, Gunning Fog Index, Fry Readability and Simple Measure of Gobbledygook (SMOG) Index. Readability was compared between academic and industry-developed PILs.

**Results:**

A total of 46 PILs were analysed in this study: 38 (82.6%) industry and 8 (17.4%) academic-led clinical trials. PILs had a mean (± SD) total word count of 8426 (± 2002). The overall mean readability scores, and equivalent reading ages were as follows: Flesch Reading Ease: 53.5 – age 15 + ; Flesch-Kincaid Grade Level: 9.9 – age 14–15; Gunning Fog Index: 11.6 – age 16–17; Fry Readability: 9.9 – age 14–15; SMOG: 13.5 – age 19 + . Academic PILs were more readable than industry PILs, with statistically significant differences for the Flesch Reading Ease (p = 0.046), Gunning Fog Index (p = 0.035), Fry Readability (p = 0.041), and SMOG Index (p = 0.010). No significant difference was observed for the Flesch–Kincaid Grade Level.

**Conclusions:**

All PILs were written at a reading age far above recommended levels, raising concerns that many potential participants, particularly those with lower literacy or living in areas of higher deprivation, may struggle to understand essential trial information or may be deterred from taking part altogether. While readability formulas capture only one dimension of comprehensibility, these findings highlight an urgent need to redesign PILs to reduce reading age and to evaluate how improving readability impacts participant understanding and informed consent.

## Background

Health literacy has recently been described as “linked to literacy, and entails people's knowledge, motivation and competences to access, understand, appraise, and apply health information in order to make judgments and take decisions in everyday life concerning healthcare, disease prevention and health promotion to maintain or improve quality of life during the life course” [1]. It is reported that up to half of the population have limited health literacy [2]. Although health literacy is more than simply the ability to read and understand words, up to 61% of the working age population in England find health materials difficult to understand due to their complexity and 1 in 6 adults (16.4%) have very low levels of literacy skills. In the UK it is estimated that 7.1 million adults read and write at or below the level of a nine-year-old [3, 4].

Readability, the ease with which a reader can understand a written text, directly affects how well people can access, understand, and use written health information to make informed health decisions. If written materials are too complex (e.g., long sentences, jargon), people with limited literacy may not understand key health messages. This acts as a barrier to their ability to access and apply information when making decisions about their healthcare and contributes to low health literacy. Lower levels of health literacy are more prevalent within economically and socially disadvantaged communities, including people with learning disabilities or those with language barriers, which can widen health inequalities demonstrating the link between literacy and life expectancy [4].

The NHS (UK) recommends that written healthcare materials should be aimed at the readability stage expected of the average 11 year old [5], similarly in the US, the Agency for Healthcare Research and Quality recommends a 5th or 6th grade level (age 10–12) [6].

Taking part in early phase clinical trials is a complex healthcare decision and requires a thorough understanding of the potential risks and benefits of participation. One of the key principles of ethics in clinical research codified in the Declaration of Helsinki is autonomy [7]. This fundamental ethical principle refers to the right and ability of a participant to make independent, informed decisions and is implemented through the practice of ‘informed consent’. Prior to providing consent to participate in a clinical drug trial, potential participants are provided with written information about the drug in the form of a standardised participant information leaflet (PIL). This written document is the primary source of information that participants will review as part of the decision-making process and in most cases will be an individual’s initial introduction to the trial. Considering the significance of the PIL, it should be clear and accessible, allowing the recipient to understand it fully. These documents should promote comprehension by using simple language and concise sections to explain drug risks and address any questions about medication use. It is essential to develop PILs that deliver clear and accurate information to safeguard an individual’s health and to enable them to have an understanding about the risks of taking part in the specific trial [8, 9].

The NIHR Liverpool Clinical Research Facility (CRF) is a phase I clinical research facility based within Liverpool University Hospitals NHS Foundation Trust with a catchment area for clinical trial recruitment across Liverpool City Region (LCR). LCR has some of the lowest literacy levels in England. Walton (Liverpool), Knowsley, West Derby (Liverpool) and Birkenhead are in the top 28 constituencies with the greatest literacy need. This increases the likelihood of potential participants being unable to understand documentation presented to them [10]. Phase I is the critical dose-determining step in the drug development pipeline; the focus is safety and tolerability and is mostly undertaken in healthy volunteers. There is usually very little, if any, human experience data contained within the PILs, and complex information about animal models and in vitro studies needs to be explained. These are particularly challenging scientific concepts, which require clear and understandable explanation if participants are to make informed decisions about participation.

Readability of a document can be assessed using readability formulas, which evaluate the reading age required to understand a document, through the analysis of sentences, paragraphs and word length. They are commonly used in health settings to determine the understandability of information [11]. There are various formulas in use such as Flesch Reading Ease, Gunning Fog Index and SMOG (Simple Measure of Gobbledygook) Index. These formulas utilise different calculations and validation methods; therefore, the results of each formula may differ. The use of readability scores to assess the complexity of written information in participant information leaflets may aid in the writing of PILs that use language that is understandable to all participants [12].

Many studies examining readability of clinical trial information have been done for later phase studies of effectiveness, in which there is existing data on safety from human participants. This has also been examined in cancer and surgical trials, in which the trial paradigm is quite distinct [13–15]. To the authors’ knowledge, this is the first study in adults to examine readability of PILs specifically in phase I clinical trials.

This study aims to explore the readability scores and reading age of PILs for recent phase I clinical trials carried out in the CRF and whether they are written at an appropriate reading age for the target population.

## Methods

### Setting

The study was a single-centre study undertaken at NIHR Liverpool Clinical Research Facility, within The Royal Liverpool University Hospital, Liverpool, UK. This study formed part of a wider programme of work aimed at improving inclusivity in early phase clinical trials.

### Eligible studies

All industry and academic-led phase I clinical trials (n = 46) that had been conducted within the Liverpool Clinical Research Facility (CRF) during a 5-year period (2017–2022) were included in the study. Phase II-IV studies and phase I trials conducted outside of the CRF were excluded.

### Regulatory approvals

In line with HRA guidance at the time of writing, ethical approval was not necessary for this study as it comprised a review of existing documentation.

### Readability software and data collection

The 5 readability formulas most used to review written health information were used to test the readability of all PILs [12]. These were Flesch Reading Ease (FRE), Flesch-Kincaid Grade Level (FKGL), Gunning Fog Index (GFI), Fry Readability and Simple Measure of Gobbledygook (SMOG) Index Table 1 summarises the scores, advantages and disadvantages of each of the formula. An online tool was used to access these readability formulas (readable.com). The content of each PIL was converted to a text file, with headings, tables and any references or appendices removed before analysis.

**Table 1:** A summary of the interpretation of score, advantages and disadvantages of each of the reading formula used in the research (Adapted from [16] and [17])

Reading formula	Understanding the score	Advantages	Disadvantages
Flesch Reading Ease	The higher the score the lower the reading age	Well established and available as part of the MS Office Software	Based on word and sentence length. Health information contains some complex concepts and to lower the score by reducing word length may lose some of the meaning
Flesch-Kincaid Grade Level	The score relates to the American school grade therefore the lower the score the lower the reading age	Well established and easily accessible as is available as a function in word-processing packages such as MS Word	Reduced accuracy when evaluating materials intended for low-level literacy
Gunning Fog Index	The score relates to the American school grade therefore the lower the score the lower the reading age	Results easy to interpret	All words with syllables of 3 or more are counted as complex, even if used in everyday language
Fry Readability	The score relates to the American school grade therefore the lower the score the lower the reading age	Used across multiple disciplines and easy to calculate scores	Unpredictable results and the potential to be unable to calculate readability score
SMOG Index	The score relates to the American school grade therefore the lower the score the lower the reading age	Used widely in healthcare due to its ease of use and consistency in results and widely validated in health literature	Unable to use in languages other than English

### Data interpretation and statistical analysis

The readability formulas generate a score that corresponds to the American educational system either as a grade or title; Table 2 shows the breakdown of these grades and titles in comparison to the corresponding age and UK school year.

**Table 2:** Breakdown of American educational levels against age and the comparable UK School year. (Adapted from ACS International School [18])

Title	American School Grade	UK School Year	Age
Pre-school	P	Nursery Pre-school (F1)	3–4
Pre-Kindergarten	Pre-K	Reception (F2)	4–5
Kindergarten	K	Year 1 (Key Stage 1)	5–6
First Grade	1	Year 2 (Key Stage 1)	6–7
Second Grade	2	Year 3 (Key Stage 2)	7–8
Third Grade	3	Year 4 (Key Stage 2)	8–9
Fourth Grade	4	Year 5 (Key Stage 2)	9–10
Fifth Grade	5	Year 6 (Key Stage 2)	10–11
Sixth Grade	6	Year 7 (Key Stage 3)	11–12
Seventh Grade	7	Year 8 (Key Stage 3)	12–13
Eighth Grade	8	Year 9 (Key Stage 3)	13–14
High School Freshman	9	Year 10 (Key Stage 4)	14–15
High School Sophomore	10	Year 11 (Key Stage 4)	15–16
High School Junior	11	Year 12 (Lower 6th)	16–17
High School Senior	12	Year 13 (Upper 6th)	17–18
College Freshman	13	University Year 1	19
College Sophomore	14	University Year 2	20
College Junior	15	University Year 3	21
College Senior	16	N/A	22 +

A comparison was made between the PIL readability scores according to whether they were developed by Industry (n = 38) or Academic (n = 8) partners. Significance was assessed using Welch’s t-test, allowing for unequal sample size and variance. A *p-*value of < 0.05 was used to determine statistical significance. The data were consistent normally distributed as observed through measurement of kurtosis and skewness using Microsoft Excel. Cohen’s d was used to determine the effect size.

## Results

### Eligible studies

There were 46 phase I clinical trials identified during the period of 2017–2022. For each of these clinical trials an electronic copy of the participant information leaflet was acquired. The target population was healthy volunteers (n = 5), patients (n = 27) and hybrid trials involving both (n = 14). There were 38 (82.6%) industry and 8 (17.4%) academic-led clinical trials.

### Readability scores

Overall, the eligible PILs had an overall mean (± SD) total word count of 8426 (± 2002). The word counts ranged from 2343 to 12,888 words.

The mean (± SD) readability scores for all PILs, across the various readability formulas, are shown in Table 3. The mean reading age calculated by the readability formulas ranged from 14–19 years, well above the commonly recommended reading age of 11 years for public-facing health information. The lowest reading age across the PILs was 13–14 years (grade 8).

**Table 3:** Mean readability scores across all PILs, including comparison between academic-developed and industry-developed PILs

	**All PILs (Mean and SD)**	**Overall Reading Age**	**Academic-developed PILs (Mean and SD)**	**Industry-developed PILs (Mean and SD)**	**p value**	**Cohen’s d**
Flesch Reading Ease	53.5 (± 3.2)	14 +	55.9 (± 3.3)	53.0 (± 3.1)	p = 0.046*	−0.9
Flesch-Kincaid Grade Level	9.9 (± 0.7)	14–15	9.5 (± 0.7)	10.0 (± 0.7)	p = 0.090	0.7
Gunning Fog Index	11.6 (± 0.8)	16–17	11.0 (± 0.8)	11.7 (± 0.8)	p = 0.035*	0.9
Fry Readability	9.9 (± 0.8)	14–15	9.4 (± 0.7)	10.1 (± 0.8)	p = 0.041*	0.9
SMOG Index	13.5 (± 1)	19 +	12.8 (± 0.7)	13.7 (± 1.0)*	p = 0.010	0.9
Word Count	8426 (± 2002)	N/A	6780 (± 2568)	8772 (± 1708)	p = 0.070	1.1

* p = < 0.05, indicates statistically significant difference in readability score between academic and industry developed PILs, Cohen’s d is a measure of effect size in the difference in readability score between academic and industry developed PILs, where approximately 0.8 indicates a large effect. ‘Overall reading age’ is based on the readability scores values from ‘all PILs’

### Comparison of the readability of PIL according to industry or academic-led study

Sponsor type was examined because differences in document development processes, institutional templates, legal review, and regulatory interpretation between academic and industry sponsors may influence the readability and accessibility of participant information leaflets. Industry-sponsored studies may involve more extensive legal and risk-related content which may affect readability.

Table 3 shows mean readability scores in all PILs and compares those developed with academic and industry partners respectively. When assessed as a group across all measures, academic-developed PILs demonstrated better readability than industry-developed PILs, with lower mean reading ages in three of the readability formulas and lower mean word counts. All PILs were found to be above the recommended reading age regardless of origin. The SMOG Index, which emphasises polysyllabic word use and is considered a robust measure of health-related readability, was the formula that gave the highest mean reading age across all PILs.

There was a statistically significant difference when four of the five readability scores of academic-developed PILs and industry-developed PILs were compared: Flesch Reading Age (p = 0.046), Gunning Fog Index (p = 0.035), Fry Readability (p = 0.041) and SMOG Index (p = 0.010). This suggests that industry-developed PILs may be harder to read and understand, which is important as the majority of phase I studies are developed by industry sponsors.

## Discussion

The Health Education England health literacy guide recommends written material to be aimed at the average 11 year old [5]. We have therefore used a reading age of 11 (equivalent to US grade 5) as the cut off for an acceptable readability score. This equates to a score 5 in all readability scores used in this study apart from FRE, where the equivalent score is 80 + [19, 20]. There is evidence that healthcare documentation, including informed consent forms (ICFs) for clinical trials, is written to a level exceeding the national average reading age [21].

Our data show that all the phase I research studies analysed in this study have PILs with readability scores that exceed the recommended reading age, despite recruiting from a region with lower than the national average reading age. Informed consent for trial participation relies on participants being able to understand the information contained within PILs [14]. The results of this study show that for phase I clinical trials, arguably the most complex and uncertain part of the clinical trial pipeline, this information is not being adequately provided in PILs.

Readability scores are calculated using factors such as the number of syllables, characters and sentence length to produce the estimated grade level of the text for the general population [22, 23]. These scores don’t provide an understanding of comprehensibility, which may involve a range of broader cognitive and contextual skills. For example, some participants in disease specific trials will be experts by experience and may have a greater understanding of medical terminology and treatment information.

PILs are just one aspect of an informed consent process that is often complex. The findings of this study present ethical implications specifically with respect to autonomy; individuals need to make voluntary decisions based on adequate understanding of trial elements including the risks, benefits, alternative treatment options and trial processes they will be exposed to [24]. It is widely understood that there is a link between low health literacy and socio-economic disadvantages; therefore, PILs with high readability scores can systematically exclude disadvantaged communities, contributing to inequality [25].

To ensure truly informed consent, more needs to be done to facilitate the understanding of complex written materials for individuals participating in research. Readability tools are now widespread and integrated into basic word processing software and provide guidance as to what can be changed to improve readability. The advent of artificial intelligence (AI) software, in particular generative AI and large language models (LLMs) could also be a powerful tool for achieving this during the design stage. A recent study examining patient education materials for aortic stenosis demonstrated that two AI-powered LLMs, ChatGPT-3.5 and Bard, were able to successfully convert materials from 10th to 12th grade level down to 6th–7th grade level [26]. However, it has also been shown that AI-generated information leaflets have omitted important information or over-simplified information resulting in a negative effect on comprehension [27].

Although studies have examined the readability of PILs, less is known about the relationship between readability of written material and its effect on comprehension. Demonstrating that improving readability of PILs can improve comprehension, is essential to ensuring that changes are made to the way PILs are written to enable greater accessibility.

## Strengths and limitations

To our understanding this is the first time readability of phase I PILs has been conducted across a wide range of specialities, over a period of 5 years, The results support similar findings across other trial literature.

Our work does have some limitations. When calculating the reading age, only one readability website (readable.com) was used to access multiple calculators. To make the findings more robust, the use of an additional calculator may be beneficial. Some research has used two websites; however, the differences in the resulting readability scores between multiple calculators are small [28].

Readability scores calculate reading age only, and do not determine whether a participant has fully understood the written information. Research is currently being conducted at NIHR Liverpool CRF, to establish whether reducing the reading age of a document results in greater comprehension.

## Conclusions

It is vital for PILs to be understandable to all potential participants. The phase I PILs included in this study did not meet existing recommendations around readability. There are existing and emerging tools that may help improve readability of PILs, AI and LLM powered software may be a tool to support improved readability, however care needs to be taken to ensure accuracy and accountability. Readability formulas are only one aspect that should be considered when assessing written health information. Other measures to be considered include co-producing with public contributors or patients with lived experience or presenting information in different formats including audio and video formats. Comprehension and application of written health information is ultimately what contributes to health literacy and allows for valid informed participation in early phase trials. Future work may focus on assessing whether reducing the reading age supports an increase in overall comprehension and meaningful informed consent.

## Data Availability

The datasets generated and/or analysed during the current study are not publicly available due to confidentiality but are available from the corresponding author on reasonable request.

## Abbreviations

AI: Artificial Intelligence
CRF: Clinical Research Facility
FKGL: Flesch-Kincaid Grade Level
FRE: Flesch Reading Ease
GFI: Gunning Fog Index
LCR: Liverpool City Region
LLM: Large Language Model
NIHR: National Institute for Health & Care Research
PIL: Participant Information Leaflets
SMOG: Simple Measure Of Gobbledygook
TherEx: Centre for Experimental Therapeutics
UK: United Kingdom

## References

[1] Sorensen K, et al. Health literacy and public health: a systematic review and integration of definitions and models. BMC Public Health. 2012;12:80. 10.1186/1471-2458-12-80 PMC3292515 22276600

[2] Teravainen-Goff A, et al. Seldom-heard voices: Adult literacy in the UK. 2022. Cited 1 Nov 2025; Available from: https://cdn.literacytrust.org.uk/media/documents/Adult_Literacy_2022_report_FINAL.pdf.

[3] NIHR. Health information: are you getting your message across? 2022 Cited 2025 14/2/2025; Available from: https://evidence.nihr.ac.uk/collection/health-information-are-you-getting-your-message-across/.

[4] National Literacy Trust. Adult literacy. n.d. Cited 2024 25/10/2025; Available from: https://literacytrust.org.uk/parents-and-families/adult-literacy/.

[5] Health Education England. Health literacy ‘how to’ guide. n.d. Available from: https://library.nhs.uk/wp-content/uploads/sites/4/2020/08/Health-literacy-how-to-guide.pdf

[6] Brach C. AHRQ health literacy universal precautions toolkit. Agency for Healthcare Research and Quality; 2004.

[7] World Medical Association Declaration of Helsinki - ethical principles for medical research involving human participants. 2004. Available from: https://www.wma.net/policies-post/wma-declaration-of-helsinki/10.1001/jama.2024.21972 39425955

[8] HRA. Consent and participant information guidance. 2020 Cited 2024 27/10/2024; Available from: https://www.hra-decisiontools.org.uk/consent/docs/Consent%20and%20PIS%20Guidance.pdf

[9] Dwyer CP, et al. Comparing the effects of a patient-designed-and-informed participant information leaflet in comparison with a standard, researcher-designed information leaflet on recruitment, retention and understanding: a study-within-a-trial. Contemp Clin Trials Commun. 2022;28:100936. 10.1016/j.conctc.2022.100936 PMC9192790 35711678

[10] Experian, Identifying the places with the greatest literacy needs: constituencies in England ranked by need. 2016, Experian. Cited: 1 Nov 2025; Available from: https://cdn.literacytrust.org.uk/media/documents/Constituency_table_-_by_rank_-_FINAL.pdf.

[11] Ley P, Florio T. The use of readability formulas in health care. Psychol Health Med. 1996;1(1)(1996–02):7–28.

[12] Wang LW, et al. Assessing readability formula differences with written health information materials: application, results, and recommendations. Res Social Adm Pharm. 2013;9(5):503–16. 10.1016/j.sapharm.2012.05.009 22835706

[13] Grundner TM. How to test the readability of your surgical consent form. Same Day Surg. 1980;4(8):65–9. 10317001

[14] Morrow GR. How readable are subject consent forms? JAMA. 1980;244(1):56–8. 7382056

[15] Terblanche M, Burgess L. Examining the readability of patient-informed consent forms. Open Access J Clin Trials. 2010;2010(2):157–62.

[16] Readable. Readability formulas. 2011 Cited 2025 01/04/2025; Available from: https://readable.com/readability/readability-formulas/.

[17] Nash E, et al. The readability of parent information leaflets in paediatric studies. Pediatr Res. 2023;94(3):1166–71. 10.1038/s41390-023-02608-z PMC10444605 37120650

[18] ACS International School. Grade equivalent chart. 2024 Cited 2025 01/01/2025; Available from: https://www.acs-schools.com/egham/wp-content/uploads/sites/3/2024/09/ACS-Egham-Grade-Equivalence-2024.pdf.

[19] Wylie Communications. How to calculate readability with the Flesch Reading Ease, Flesch-Kincaid and Gunning Fog tests. 2026 15 June 2026]; Available from: https://www.wyliecomm.com/measure-reading-levels-with-readability-indexes/.

[20] Flesch R. A new readability yardstick. J Appl Psychol. 1948;32(3):221–33. 10.1037/h0057532 18867058

[21] Mirza FN, et al. The literacy barrier in clinical trial consents: a retrospective analysis. EClinicalMedicine. 2024;75:102814. 10.1016/j.eclinm.2024.102814 PMC11701435 39763593

[22] Singh S, Jamal A, Qureshi F. Readability metrics in patient education: where do we innovate? Clin Pract. 2024;14(6):2341–9. 10.3390/clinpract14060183 PMC11586978 39585011

[23] Szabo P, Biro E, Kosa K. Readability and comprehension of printed patient education materials. Front Public Health. 2021;9:725840. 10.3389/fpubh.2021.725840 PMC8670754 34917569

[24] Burks A.C. Keim-Malpass J., Health literacy and informed consent for clinical trials: a systematic review and implications for nurses. Nursing: Res Rev. 2019. page 31-40 10.2147/NRR.S207497.

[25] Schillinger D. The intersections between social determinants of health, health literacy, and health disparities. Stud Health Technol Inform. 2020;269:22–41. 10.3233/SHTI200020 PMC7710382 32593981

[26] Rouhi AD, et al. Can artificial intelligence improve the readability of patient education materials on aortic stenosis? A pilot study. Cardiol Ther. 2024;13(1):137–47. 10.1007/s40119-023-00347-0 PMC10899139 38194058

[27] Verran C. Artificial intelligence-generated patient information leaflets: a comparison of contents according to British Association of Dermatologists standards. Clin Exp Dermatol. 2024;49(7):711–4. 10.1093/ced/llad461 38169318

[28] Erkin Y, et al. Readability, quality, and reliability of AI-generated ınformation on myofascial pain syndrome: A comparative analysis of ChatGPT, Gemini, and Perplexity. 2026. 10.1371/journal.pone.0350402. PMC13235886 42241388

